# B4GALNT2 and xenotransplantation: A newly appreciated xenogeneic antigen

**DOI:** 10.1111/xen.12394

**Published:** 2018-03-31

**Authors:** Guerard Byrne, Saadullah Ahmad‐Villiers, Zeji Du, Christopher McGregor

**Affiliations:** ^1^ Institute of Cardiovascular Science University College London London UK; ^2^ Department of Surgery Mayo Clinic Rochester MN USA

**Keywords:** antibody‐mediated rejection, beta‐1,4‐N‐acetyl‐galactosaminyltransferase 2, cardiac xenotransplantation, genetic modification, xenotransplantation

## Abstract

Analysis of non‐Gal antibody induced after pig‐to‐baboon cardiac xenotransplantation identified the glycan produced by porcine beta‐1,4‐N‐acetyl‐galactosaminyltransferase 2 (B4GALNT2) as an immunogenic xenotransplantation antigen. The porcine B4GALNT2 enzyme is homologous to the human enzyme, which synthesizes the human SDa blood group antigen. Most humans produce low levels of anti‐SDa IgM which polyagglutinates red blood cells from rare individuals with high levels of SDa expression. The SDa glycan is also present on GM2 gangliosides. Clinical GM2 vaccination studies for melanoma patients suggest that a human antibody response to SDa can be induced. Expression of porcine B4GALNT2 in human HEK293 cells results in increased binding of anti‐SDa antibody and increased binding of *Dolichos biflorus* agglutinin (DBA), a lectin commonly used to detect SDa. In pigs, B4GALNT2 is expressed by vascular endothelial cells and endothelial cells from a wide variety of pig backgrounds stain with DBA, suggesting that porcine vascular expression of B4GALNT2 is not polymorphic. Mutations in B4GALNT2 have been engineered in mice and pigs. In both species, the B4GALNT2‐KO animals are apparently normal and no longer show evidence of SDa antigen expression. Pig tissues with a mutation in B4GALNT2, added to a background of alpha‐1,3‐galactosyltransferase deficient (GGTA1‐KO) and cytidine monophosphate‐N‐acetylneuraminic acid hydroxylase deficient (CMAH‐KO), show reduced antibody binding, confirming the presence of B4GALNT2‐dependent antibodies in both humans and non‐human primates. Preclinical xenotransplantation using B4GALNT2‐deficient donors has recently been reported. Elimination of this source of immunogenic pig antigen should minimize acute injury by preformed anti‐pig antibody and eliminate an induced clinical immune response to this newly appreciated xenotransplantation antigen.

**Abbreviations**
ANXA2: Annexin A2
B4GALNT2: beta‐1,4‐N‐acetyl‐galactosaminyltransferase 2
CMAH: cytidine monophosphate‐N‐acetylneuraminic acid hydroxylase
DBA: *Dolichos biflorus* agglutinin
EC: endothelial cell
Gal: galactose alpha 1,3, galactose
GalNAc: N‐acetylgalactosamine
GGTA‐1: alpha‐1,3‐galactosyltransferase
GI: gastrointestinal
GTKO: GGTA1 mutant
Neu5Gc: N‐acetylneuraminic acid
NHP: non‐human primates
PBMC: peripheral blood mononuclear cell
PROC: endothelial cell protein C receptor
RBC: red blood cell
TACA: tumor‐associated carbohydrate antigen
THGP: Tamm‐Horsfall glycoprotein
XTx: xenotransplantation

## 1 INTRODUCTION

Xenotransplantation (XTx) is limited by recalcitrant antibody‐mediated rejection occurring either hyperacutely immediately after transplant (HAR) or at later times, referred to as delayed xenograft rejection.^1^, ^2^, ^3^ These rejection mechanisms result from the abundance of human and non‐human primate (NHP) antibodies directed to the classic xenogeneic antigen galactose alpha 1,3 galactose (Gal) which leads to chronic or induced antibody‐mediated vascular endothelial cell (EC) injury or activation.^4^, ^5^ Gal is not expressed in humans or Old World NHPs but is expressed at high levels in porcine tissues. Genetically modified pigs, with a mutation in the GGTA‐1 locus (GTKO), do not express the Gal antigen.^6^ The introduction of GTKO donor organs eliminated anti‐Gal‐mediated xenograft rejection, but did not eliminate antibody‐mediated rejection and instead highlighted the importance of antibody directed to non‐Gal pig antigens.^7^, ^8^

Non‐Gal antibody in human and NHP serum is reactive to both protein and carbohydrate antigens.^9^, ^10^, ^11^, ^12^, ^13^ The currently identified immunogenic EC carbohydrate antigens include Gal, glycans modified with N‐glycolylneuraminic acid (Neu5Gc), and a carbohydrate antigen (SDa) produced by the porcine β1,4 N‐acetylgalactosaminyltransferase‐2 (B4GALNT2). The human blood group A antigen is also potentially immunogenic^14^; however, high rates of A‐type blood group polymorphism in the pig permit exclusion of this antigen by selective breeding.^15^, ^16^ Collectively, antibody reactivity to the 3 major xenogeneic glycans, Gal, Neu5Gc‐modified glycans, and SDa, accounts for the majority of preformed human anti‐pig antibody reactivity.^17^, ^18^

Humans, but not NHPs, make an array of antibodies to Neu5Gc‐modified glycans.^19^, ^20^ This antibody reactivity is expected to contribute to clinical xenograft rejection, but determining its impact remains difficult due to the absence of anti‐Neu5Gc antibody in experimental NHP models. There have been excellent recent reviews on Neu5Gc and the potential immunogenicity of Neu5Gc‐modified glycans in XTx.^21^, ^22^, ^23^ Less is known about the expression and immunogenicity of the glycan produced by porcine B4GALNT2. The clinical contribution of B4GALNT2 and SDa, the glycan it synthesizes, has been recently reviewed.^24^ The purpose of this review is to summarize the current experimental and clinical information on B4GALNT2 gene expression and the SDa antigen, with an emphasis of its potential impact on future clinical use of porcine organs and XTx.

## 2 THE SDA HUMAN BLOOD GROUP

The SDa blood group, synthesized by the human B4GALNT2 glycosyltransferase, was independently identified 50 years ago by Renton et al^25^ and by Macvie et al.^26^ The initial identification was based on a small set of unrelated serum samples which agglutinated the majority of Caucasian red blood cell (RBC) samples. The degree of RBC agglutination varied widely, presenting a mixed field reaction with variably sized agglutinates against a large background of free RBCs. Further analysis, using agglutination inhibition, identified SDa+ expression in human saliva, milk, feces, and urine with more than 50% of people with SDa− RBCs being SDa+ in saliva or urine.^26^, ^27^ Based on urinary secretions, persons of European ancestry are 96% SDa+ and the frequency of SDa− individuals, lacking SDa expression on both RBCs and secretions, is about 4%.^27^

The initial descriptions of anti‐SDa antibody, based on RBC agglutination studies, indicated only 1%‐2% of individuals produced anti‐SDa IgM which weakly agglutinated SDa+ RBCs, preferentially at low temperature.^25^, ^26^ The apparent frequency of anti‐SDa antibody increased, however, when cells with stronger SDa expression are used. Clear evidence supporting the classification of SDa as a high‐frequency polyagglutinable antigen^28^ was evident with the discovery of a Mauritan family in which B‐ or O‐type RBCs were unexpectedly agglutinated by *Dolichos biflorus* agglutinin (DBA). DBA normally recognizes the N‐acetylgalactosamine (GalNAc) component of blood group A1. The target of this unusual B‐ or O‐type RBC DBA agglutination, called CAD, was subsequently shown to react strongly with anti‐SDa and some non‐CAD, O‐type SDa+ RBCs with high SDa antigen levels were shown to bind DBA.^29^, ^30^ This suggested that CAD, also termed Sd(a++), shared a common antigen and that CAD RBCs represent an extreme high level of SDa expression. Serum from most individual agglutinates CAD RBCs,^29^, ^31^ indicating that there is commonly a low level of anti‐SDa IgM in human serum. Notably, non‐A1‐type human RBCs are not agglutinated by DBA except, as noted above, in cases of high SDa expression. Due to the generally low expression of SDa on RBC in most individuals, the rarity of CAD individuals and the low level and low‐temperature reactivity of anti‐SDa IgM, the SDa antigen, and antibody do not create a significant transfusion risk. Rare hemolytic reactions have been reported, however, in association with RBCs with high levels of SDa expression.^32^, ^33^ This suggests that porcine ECs and organs expressing SDa (discussed below) may be at risk of early antibody‐mediated immune injury.

The B4GALNT2 enzyme, isolated from various human^34^, ^35^, ^36^ and animal tissues,^37^, ^38^, ^39^, ^40^ catalyzes the synthesis of the SDa blood group antigen by the addition of beta 1,4‐linked GalNAc to an alpha 2,3 sialic acid‐modified N‐acetyl lactosamine acceptor oligosaccharide. The B4GALNT2 enzyme preferentially utilizes N‐acetylneuraminic acid (Neu5Ac) α2,3 galactose β1,4 glucose as an acceptor molecule and does not react with Neu5Ac α2,6‐modified lactose. Additional alpha 2,3 sialylated acceptor molecules that can be modified by B4GALNT2 include both type 1 and 2 lactosamine, Core 1 and 3 O‐linked glycans, and paraglobosides.^24^ For each of these, the SDa trisaccharide (Table 1) present on human red blood cells (RBCs), rare CAD RBC protein, and glycolipids,^41^, ^42^ and the major urinary mucin protein Tamm‐Horsfall glycoprotein (THGP),^43^ is the terminal B4GALNT2‐dependent structure. The SDa epitope is commonly detected by reactivity to DBA^30^, ^44^ or by anti‐SDa monoclonal antibodies.^40^, ^45^, ^46^

**Table 1:** SDa containing glycans

Carbohydrate	Source	Reference
GalNAc β1,4 (Neu5Ac α2,3) Gal β*1,4 GlcNAc* β*1,3 Gal*	RBC glycophorin, THGP	43
GalNAc β1,4 (Neu5Ac α2,3) Gal β*1,3 (Neu5Ac* α*2,6) GalNAc‐Ser/Thr* GalNAc β1,4 (Neu5Ac α2,3) Gal β*1,4 GlcNAc* β*1,3 Gal* β*1,4 Glc‐ceramide*	CAD RBCs	41, 93
GalNAc β1,4 (Neu5Ac α2,3) Gal β*1,4 Glc‐ceramide*	GM2 ganglioside	94
GalNAc β1,4 (Neu5Ac α2,3) Gal β*‐**R***	SDa trisaccharide	a

a This terminal trisaccharide structure is shared between each of the major sources of SDa antigen.

The SDa trisaccharide present on the GM2 ganglioside (Table 1) is synthesized by the distinct B4GALNT1 transferase which shows only limited homology with B4GALNT2.^47^ This is analogous to the relationship between rat GGTA‐1, which adds Gal antigen to glycoproteins, and iGB3 synthase, which adds the Gal antigen to iGb3, but not glycoproteins.^48^ Excessive accumulation of GM2 ganglioside, due to a deficiency in β‐N‐acetylhexosaminidase, contributes to ongoing cellular damage associated with Tay‐Sachs disease. Mutations in B4GALNT1 have been identified, which lead to deficiency in GM2 synthesis and are associated with a hereditary spastic paraplegia.^49^ This suggests that targeted mutation of the porcine B4GALNT1 gene, to eliminate SDa antigen on GM2 gangliosides, may have deleterious effects on donor animals. Mutation of B4GALNT1 would not affect SDa modification of glycoproteins or of other glycolipids that are present in CAD RBCs. For xenotransplantation, the utility of mutating B4GALNT1 remains uncertain as the primary immune stimulation, at least in NHPs, appears to be from B4GALNT2‐dependent antigens.

## 3 INDUCTION OF ANTI‐SDA ANTIBODY

The GM2 ganglioside is a tumor‐associated carbohydrate antigen (TACA) detected at higher levels in malignant melanoma and other forms of cancer.^50^ Significant efforts have been made to develop tumor vaccines based on TACA targets including GM2.^51^ Clinical vaccination studies with purified GM2^52^ or GM2 conjugated to keyhole limpet hemocyanin (GM2‐KLH)^53^, ^54^ have shown variable induction of cytolytic anti‐GM2 IgM and IgG. Most patients show an induced antibody response, but only about 10% show sustained induction of cytolytic IgM and IgG which react in both GM2 ELISA and whole‐cell binding (flow cytometry) assays.^53^ Other patients develop lower levels of cytolytic antibody or antibodies to epitopes not present on the cell surface. It is unclear from these studies what proportion of induced anti‐GM2 antibodies reacts specifically with the SDa trisaccharide or with the glycan ceramide or other GM2 epitopes. In an early clinical study of stage III melanoma patients, those patients receiving GM2 vaccines demonstrated an induced IgM and IgG responses and some evidence of prolonged relapse‐free survival.^55^ Subsequent phase 3 clinical studies consistently report induction of anti‐GM2 antibody but failed to show a therapeutic benefit for the primary endpoints of relapse‐free survival or overall survival.^56^, ^57^ Subset analysis, however, showed a trend for improvement in patients with high titers of anti‐GM2, suggesting that the effects of GM2‐KLH vaccination might be limited by a heterogeneous immune response. More recently, GM2 vaccine development has focused on using neoconjugates made with synthetic SDa tetrasaccharides.^58^, ^59^ These conjugates induce strong cytolytic anti‐GM2 antibody in Balb/c mice, which clearly bind the SDa glycan. Clinical GM2 vaccination studies consistently induce an antibody response, which is at least in part directed to the SDa glycan. This suggests that humans, challenged with SDa‐positive xenogeneic tissue, may induce an anti‐SDa response.

## 4 EXPRESSION OF B4GALNT2 AND SDA

Human B4GALNT2 is predominantly expressed in gastrointestinal (GI) epithelium of the colon with lower levels of expression, evident by Northern blot, in the kidney, ileum, stomach, and rectum.^47^, ^60^, ^61^ These sites also show reactivity to DBA or anti‐SDa antibody.^62^ Consistent with this analysis, B4GALNT2 enzyme has been purified from human and guinea pig kidney^36^, ^38^ and human and porcine large intestine.^35^, ^39^ There is also low expression of B4GALNT2, detected by reverse transcription PCR, in most other human tissues.^47^ This very low expression does not always correlate with detectable SDa staining; however, this does not mean that the antigen is not present as human RBCs exhibit widely variant levels of SDa, but only the highest levels of expression react with DBA.^29^, ^30^ Human mast cells, present in connective tissues, may also express B4GALNT2 as they show strong, blood group‐independent reactivity with DBA.^63^

There is a significant loss of B4GALNT2 expression in colonic tumors compared to normal tissue.^64^ The reduction of B2GALNT2 expression often correlates with a change in promoter methylation 65, 66 and with an increase in the synthesis of sialyl Lewis antigens.^60^ The increase in sialyl Lewis antigens appears to result from the reduction in B4GALNT2 expression, which alters the balance of competition between B4GALNT2 and fucosyltransferases in colonic tumors for the same acceptor substrate.^24^

Expression of B4GALNT2 and SDa in other species is chiefly characterized in mice and as a whole has not been systematically studied in other mammals. Common murine laboratory strains (BALB/c, C57BL/6) exhibit a similar GI epithelial cell‐dominated pattern of B4GALNT2 and SDa antigen expression, with a notable lack of EC expression, as seen in humans. This expression profile is broadly thought to occur in other mammals where, except for the pig, there is a consistent absence of DBA reactivity to vascular ECs.^67^ The RIII/SJ^68^ and other laboratory mouse strains which carry the modifier of von Willebrand factor‐1 (*Mvwf1*) mutation are exceptions, however, as they carry an altered regulatory domain of B4GALNT2 which causes a switch from GI epithelial to vascular EC expression.^69^, ^70^ Endothelial cell expression of B4GALNT2 in mice results in altered glycosylation of vWF, which leads to its rapid clearance from circulation and low plasma vWF levels characteristic of *Mvwf1* mice. This polymorphic expression is not unique to laboratory mice and is detected at high frequency in wild murine populations.^71^ Similar cisregulatory variations affecting B4GALNT2 expression are also present *Mus spretus* and *Mus musculus* subspecies,^72^ suggesting that a balanced GI epithelial and vascular endothelial polymorphism of B4GALNT2 expression in mice has been maintained for over 2.8 million years. The selective pressure affecting B4GALNT2 expression may derive from host pathogen interactions as the absence of B4GALNT2 expression and altered glycosylation of the intestinal epithelia is associated with significant shifts in the composition of the microbiota,^73^ which can effect host sensitivity and pathologic responses to infection.^74^ Other variations in B4GALNT2 expression are reported in ovarian follicles of FecL^L^ carrier sheep, where it is linked to increased fecundity^75^ and in respiratory tract expression in the ferret where high SDa expression reduces influenza infectivity.^76^

## 5 THE B4GALNT2 GENE IN HUMANS AND NHPS

The murine B4GALNT2 gene was isolated from a cDNA expression library made from a cytotoxic T lymphocyte cell line.^40^ This expression library was screened for reactivity to hybridoma IgM antibodies (CT1 and CT2) which bound activated cytolytic T lymphocytes, blocked target cell lysis, and reacted with the human SDa blood group antigen.^77^ The human B4GALNT2 gene was identified based on homology with the mouse gene.^47^ Both genes are composed of 11 coding exons. The murine B4GALNT2 gene produces a single transcript, whereas the human B4GALNT2 gene uniquely produces alternative splice variants resulting in a short and long form of the human protein.^47^ The long form of human B4GALNT2 includes an unusual 67 amino acid cytoplasmic domain which may affect cellular localization of this enzyme. The significance of this long isoform and its prevalence in non‐human primates remains under investigation. In both humans and mice, the encoded B4GALNT2 protein is a type II transmembrane protein containing an acidic DXD amino acid motif conserved in glycosyltransferase using UDP sugar as a donor substrate. The human protein shows 74% amino acid identity to mouse polypeptide.

Although human B4GALNT2 expression is essential for synthesis of the SDa antigen, the molecular mechanism, which affects polymorphic SDa expression in human RBCs, has not been identified. Genome‐wide analysis of copy number variation in humans, based on array comparative genomic hybridization (aCGH) or direct deep sequencing, has identified some limited structural variations and deletions in the intron between exons 3 and 4.^78^, ^79^, ^80^ These structural variations occur rarely, are not reported to impact B4GALNT2 expression and thereby appear unlikely to account for polymorphic expression of SDa. Microdeletions in chromosome 17q21.31, which occur near or include portions of the B4GALNT2 locus,^81^ cause pleiotropic developmental and neurologic effects. These microdeletions are also rare, and their effects have not been attributed to the loss of B4GALNT2 expression. Changes in DNA methylation within the B4GALNT2 promoter affect gene expression 66 and have been noted in Gl cancer cells.^65^ It is unclear whether this epigenetic mechanism contributes to normal B4GALNT2 expression and polymorphic SDa synthesis in RBCs.

Expression of B4GALNT2 in NHPs is poorly characterized. There has been a report of frequent B4GALNT2 copy number variation in the chimpanzee genome 82 which likely effects B4GALNT2 expression. In an aCGH study of human and chimpanzee DNA, Perry et al^82^ detected an approximately 68‐kb deletion of the regulatory region, including the first exon of B4GALNT2, in 22 of 26 chimpanzees studied (84%). The deletion was homozygous in 38% of the samples. The effect of this deletion on B4GALNT2 expression and SDa distribution in chimpanzees was not reported; however, the location and magnitude of the deletion would suggest a potential for altered expression or loss of B4GALNT2 function. Several additional aCGH analyses have been performed comparing humans and NHPs. An extensive comparison of human, bonobo, chimpanzee, gorilla, orangutan, gibbon, macaque, baboon, marmoset and lemur was reported by Dumas et al.^83^ This study was designed to detect duplications or loss of coding sequences and did not observe any such variation in B4GALNT2 in these species. Comparative genomic studies of human, bonobo, chimpanzee, gorilla and orangutan 84, 85, 86 have not reported additional instances of regulatory deletions in B4GALNT2 other than chimpanzee. Comparable genome screening has not been reported for baboons. The predicted Papio anubis B4GALNT2 gene (XM_021928974.1) encodes a protein with 94.6% amino acid identity to human. The current baboon genome assembly (assembly Panu_3.0), unlike the chimpanzee, does not show evidence of a regulatory deletion preceding B4GALNT2 gene. The Papio anubis B4GALNT2 5′ regulatory sequences adjacent to exon 1 of B4GALNT2 (Chr16:30698372‐30778728, assembly Panu_3.0) contain over 70 kb of sequence with a high level of homology to human chromosome 17 (Chr17:49056269‐49137578, GRCh38.p7 Primary Assembly) which overlaps the beginning of the human B4GALNT2 gene. This suggests that B4GALNT2 expression in baboon may be similar to human and that the induced immune response to B4GALNT2‐positive porcine organs seen in baboons 9 may model the immune response in future clinical XTx. Further study of B4GALNT2 expression and SDa antigen distribution in the baboon would be useful.

## 6 PORCINE B4GALNT2

The porcine B4GALNT2 gene was originally identified by screening a porcine EC cDNA library for non‐Gal antigens expressed in human HEK293 cells.^10^ Purified IgG enriched in non‐Gal reactivity, derived from pig‐to‐baboon cardiac XTx recipients, was used to identify human HEK293 cell‐expressing library‐encoded non‐Gal porcine antigens on the cell surface. Six porcine gene products were identified. Most of these non‐Gal porcine target genes corresponded to well‐known EC membrane proteins (PROC, CD9, CD46, CD59 and ANXA2). HEK293 expression of these porcine gene products is subject to HEK‐derived glycosylation, suggesting that non‐Gal baboon IgG reactivity to these cell lines is directed to porcine peptide epitopes and not carbohydrates, which would be present on all HEK293 cells. In contrast, the porcine B4GALNT2 gene is a Golgi expressed glycosyltransferase not typically expressed on the cell surface. This indicates that B4GALNT2 expression in HEK293 cells altered the glycosylation of HEK293 cell membrane proteins to create a non‐Gal carbohydrate antigen.

The porcine B4GALNT2 cDNA is derived from an 11‐exon gene and encodes a protein with 76% amino acid identity to the human protein and less than 50% amino acid identity to human B4GALNT1. Expression of porcine B4GALNT2 in HEK293 cells (HEK‐B4T) results in increased reactivity to anti‐B4GALNT2 antibody, anti‐SDa (KM694), and DBA reactivity.^9^ There is also a 20‐fold enhancement of HEK‐B4T cell, compared to HEK293 cell, sensitivity to antibody‐dependent complement‐mediated lysis using serum from cardiac XTx recipients.

**Figure 1:**
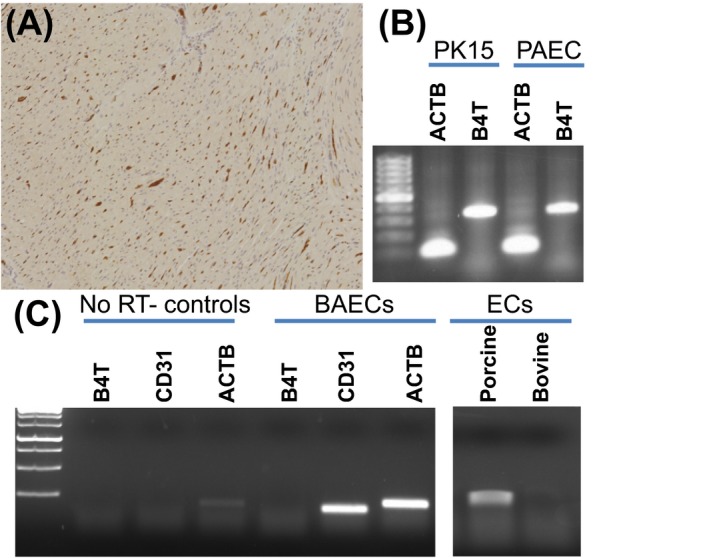
Expression of B4GALNT2 in porcine and bovine cells. A, *Dolichos biflorus* agglutinin (DBA) lectin staining of Gottingen minipig heart tissue showing capillary endothelial cell staining. B, Reverse transcriptase polymerase chain reaction analysis (RT‐PCR) of B4GALNT2 (B4T) expression in porcine PK15 and porcine aortic ECs (PAEC). RT‐PCR was performed as previously reported 9 using beta‐actin primers (ACTB, forward: CAAGATCATCGCGCCTCCA and reverse: ACTCCTGCTTGCTGATCCACATCT) and B4GALNT2 primers (B4T, forward: TACAGCCCTAGATGTCTGTC and reverse: CTCTCCTCTGAAAGTGTTCGAG). C, RT‐PCR analysis of B4GALNT2 (B4T) total RNA (400 ng/reaction) expression in bovine (BAEC) and porcine endothelial cells (EC). Primers specific for bovine B4GALNT2 (B4T, forward: CTCCAGAGCATTCGTGAGTATT and reverse: TTTGGTGGTGACCTGAGATATG), bovine beta‐actin (ACTB, forward: GTGACATCAAGGAGAAGCTCTG and reverse: AGGAAGGAAGGCTGGAAGA), and CD31 (CD31, forward: GGTCAACGTCACAGAGCTATT and reverse: CACAGTCATGCTTCCCTTCT) were used. Reactions run without a reverse transcriptase step (NO RT controls) show no gene expression. Cultured BAECs show prominent expression of CD31 but do not express B4GALNT2 (1C, left). Additional analysis (1C, right) using RT‐PCR primers conserved in B4GALNT2 in both the porcine and bovine mRNA (forward: ACAAGCTCATGACCATGCTC and reverse: TTTGGTGGTGACCTGAGATATG) detects porcine but not bovine B4GALNT2 expression. RT‐PCR for 1C was performed using one‐step RT‐PCR reaction (USB‐Affymetrix, Santa Clara, CA). Reverse transcription was performed at 42°C for 30 min, followed by 30 cycles of 95°C for 30 s, 58ºC for 30 s, and 72ºC for 50 s, and a final extension for 10 min at 72ºC. Amplification products in 1B and 1C were run in a 1.5% agarose gel

In agriculture‐based strains of pigs, mRNA for B4GALNT2 is expressed in ECs, peripheral blood mononuclear cells (PBMCs), in the GI system and major vascularized organs.^9^ Porcine EC expression of B4GALNT2 is independent of AO blood group or GGTA‐1 (GTKO) status. Pig ECs are strongly agglutinated by DBA lectin, and cultured ECs from agricultural strains, Yucatan hairless minipigs,^87^ and Panepinto micropigs^88^ have been reported to bind DBA, independent of AO blood group status. In vivo vascular B4GALNT2 EC expression, measured by DBA stain, may not be uniform in all vascular beds but is prominent in cardiac capillaries, large renal blood vessels, and glomerular ECs, in reticuloendothelial cells of the liver^9^ and ECs of the femoral artery.^89^ Cardiac capillary staining with DBA and EC expression of B4GALNT2 is also evident in Gottingen minipigs (Figure 1A,B). This is consistent with previous studies of ECs from variant porcine backgrounds and suggests that porcine EC expression of B4GALNT2 is not polymorphic and will be commonly present in most pig strains.

Porcine B4GALNT2 expression is not confined to ECs and is present in PBMCs, spleen,^9^ and epithelial cells such as PK15 (Figure 1B). Porcine primordial germ cells are also reported to express SDa and stain with anti‐SDa antibody^90^. In contrast, cultured bovine ECs do not express B4GALNT2 (Figure 1C), which is comparable to human and most murine strains, suggesting that clinical products produced from bovine tissues, such as heart valves, may be less likely to express B4GALNT2‐dependent glycans.

Porcine B4GALNT2 expression in HEK‐B4T cells results in increased anti‐SDa (KM694) and DBA reactivity; however, porcine ECs, which are strongly agglutinated by DBA, do not bind this anti‐SDa monoclonal antibody.^9^ A similar disparity between lectin and antibody binding was reported for ovine glycoproteins secreted by granulosa cells.^75^ The strict specificity of KM694, compared to DBA, suggests that on porcine cells, variation in the structure or presentation of the glycan(s) produced by porcine B4GALNT2 may occur. What is clear is that NHP non‐Gal antibody reactivity to HEK‐B4T cells can be eliminated by pre‐absorption with porcine ECs but not by human ECs, which do not express B4GALNT2 or the SDa antigen.^9^ This indicates that the porcine EC and HEK‐B4T cells share a common, B4GALNT2‐dependent, non‐Gal antigen. In NHPs after cardiac XTx with minimal immune suppression, non‐Gal antibody with preferential binding to HEK‐B4T cells, compared to control HEK cells, is consistently induced.^10^ This immune response is also evident in GTKO pig‐to‐baboon cardiac XTx recipients with full immune suppression, when a non‐Gal antibody response, determined by antibody reactivity to GTKO pig ECs, is also present.

## 7 MUTATIONS IN B4GALNT2

Mutations in B4GALNT2 have been produced in mice (John Lowe, Consortium of Functional Glycomics, 69) and pigs.^18^ The mutant mice are generally healthy and show no histological changes to the major organs but do show variations in the level of neutrophils, plasma cells, and some T‐cell subsets.^24^ Mutant mice lack DBA staining in GI epithelia and exhibit changes in microfloral composition.^73^ The loss of B4GALNT2 expression in the GI is associated with greater resistance to Salmonella infection.^74^ Mutations in B4GALNT2 in the pig have been reported, but only in conjunction with mutations in GGTA‐1 and CMP‐Neu5Ac hydroxylase (CMAH).^17^, ^18^ In these triple knockout pigs (GGTA‐1‐KO, CMAH‐KO, and B4GALNT2‐KO), loss of porcine B4GALNT2 gene eliminates DBA lectin binding to pig PBMCs, RBCs, and renal microvascular ECs^91^, ^92^ demonstrating the dependence of DBA binding on B4GALNT2 activity. The addition of the B4GALNT2 mutation to the GTKO, CMAH‐KO background, also significantly reduces the level of human non‐Gal IgM and IgG binding to pig PBMCs, confirming the presence of human antibody which binds to the porcine glycan(s) produced by the B4GALNT2 gene. As noted above, the presentation of antigen may differ when porcine B4GALNT2 is expressed in human and porcine cells, but the dependence of human antibody reactivity to B4GALNT2 expression clearly indicates that the SDa trisaccharide, present in both SDa and CAD RBCs, is likely the primary B4GALNT2‐dependent antigenic determinant.

This combination of porcine mutations, however, may have unexpected effects on the glycome which could affect human antibody reactivity in unexpected ways. This may contribute to the rather large apparent reduction in human anti‐SDa IgG binding to these cells, as IgG is not a frequent isotype for human anti‐SDa antibody. The triple knockout pigs are apparently healthy but, to our knowledge, have only been produced through nuclear transfer technology.

## 8 CONCLUSIONS

The SDa blood group, produced by the B4GALNT2 enzyme, is a recently appreciated xenogeneic antigen. The SDa glycan is commonly expressed in human GI epithelial cells and at widely variant levels in human RBCs and other tissues and fluids. Despite this antigen expression, most humans make low levels of cold reactive anti‐SDa IgM making SDa a polyagglutinable red cell antigen. Recent genetic engineering of the porcine B4GALNT2 locus confirms the presence of preformed NHP and human antibody to B4GALNT2‐dependent antigens. Although the SDa blood group is not a significant transfusion risk, the expression of B4GALNT2 in porcine ECs, the induced antibody response seen in NHPs and the results of cancer vaccination studies suggest that B4GALNT2‐dependent pig glycans may be immunogenic in humans. The major known xenogeneic glycans (Gal, Neu5Gc‐modified glycans, and SDa) account for the majority of preformed human anti‐pig antibody reactivity, with residual antibody reactivity apparently binding to a restricted set of SLA antigens.^17^ There remains, however, the possibility of additional immunogenic glycans and proteins. Identifying the B4GALNT2‐dependent non‐Gal antigens using serum from XTx recipients with minimal immune suppression underscores the utility of such transplants and suggests that future similar studies using “triple knockout” glycan‐depleted donor organs will be useful for further identification of residual preformed and induced non‐Gal antibody specificities.

## AUTHOR CONTRIBUTIONS

Dr. Byrne was responsible for drafting and revision of the manuscript, research design, and data acquisition. Dr Ahmad‐Villiers and Dr Du contributed to data acquisition, analysis, and interpretation. Dr. McGregor contributed to the concept and design of the research. All authors provided the final approval for the manuscript.

## CONFLICTS OF INTEREST

The authors declare no conflict of interest.
